# Effect of B12 and folate deficiency in hypomethylation of Angiotensin I converting enzyme 2 gene and severity of disease among the acute respiratory distress syndrome patients

**DOI:** 10.1002/jcla.24846

**Published:** 2023-03-06

**Authors:** Reza Najafipour, Davood Mohammadi, Abdolmabood Momeni, Sahar Moghbelinejad

**Affiliations:** ^1^ Genetics Research Center the University of Social Welfare and Rehabilitation Science Tehran Iran; ^2^ Department of Surgery, School of Medicine Qazvin University of Medical Sciences Qazvin Iran; ^3^ Khatam Pathobiology and Genetic Lab Qazvin Iran; ^4^ Research Institute for Prevention of Non‐Communicable Diseases, Cellular and Molecular Research Centre Qazvin University of Medical Sciences Qazvin Iran

**Keywords:** ACE‐2, B vitamins, COVID‐19, expression regulation, hypomethylation

## Abstract

**Background:**

Angiotensin I converting enzyme 2 (ACE‐2) is the most important receptor and has important role in the entry of corona virus to the host cells. The present study aimed to investigate the different mechanisms involved in the expression regulation of this gene among the COVID‐19 patients.

**Methods:**

A total of 140 patients with COVID‐19 (*n* = 70 mild COVID‐19, *n* = 70 ARDS) and 120 controls were recruited. The expression of ACE‐2 and miRNAs was evaluated by quantitative real‐time PCR (QRT‐PCR), and methylation of CpG dinucleotides in the ACE2 promoter was quantified using bisulfite pyro‐sequencing. Finally, different polymorphisms of the ACE‐2 gene were studied by Sanger sequencing.

**Results:**

Our results showed a significant high expression of the ACE‐2 gene in the blood samples of acute respiratory distress syndrome (ARDS) patients (3.8 ± 0.77) in comparison with controls (0.88 ± 0.12; *p* < 0.03). The methylation rate of the ACE‐2 gene in ARDS patients was 14.07 ± 6.1 compared with controls (72.3 ± 5.1; *p* < 0.0001). Among the four studied miRNAs, only miR200c‐3p showed significant downregulation in ARDS patients (0.14 ± 0.1) in comparison with controls (0.32 ± 0.17; *p* < 0.001). We did not see a substantial difference in the frequency of rs182366225 C>T and rs2097723 T>C polymorphisms between patients and controls (*p* > 0.05). There was a significant correlation between B12 (*R* = 0.32, *p* < 0.001), folate (*R* = 0.37, *p* < 0.001) deficiency, and hypo‐methylation of the ACE‐2 gene.

**Conclusion:**

These results for the first time indicated that among the different mechanisms of ACE‐2 expression regulation, its promoter methylation is very crucial and can be affected by factors involved in one‐carbon metabolisms such as B9 and B12 vitamins deficiency.

## Graphical abstract

ACE‐2 ovaerexperssion mechanisms in COVID‐19 patients.

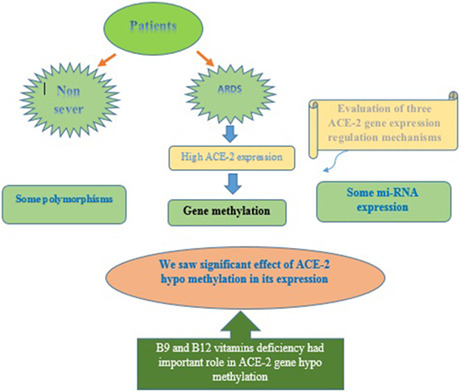

## 1 INTRODUCTION

Coronavirus disease (COVID‐19) has become a pandemic today. All researchers from all over the world are conducting numerous studies to treat this disease, while the definitive cure for this disease is not yet known. The disease is caused by severe acute respiratory syndrome coronavirus 2 (SARS‐CoV‐2).^1^ Patients with severe symptoms usually show an immune system storm, and one of the organs involved is the lung, which eventually leads to respiratory distress.^2^ Studies have shown that different molecular mechanisms are involved in the hosts' response to the virus. The virus generally enters the host cell in five ways: adhesion, penetration, biosynthesis, maturation, and release. SARS‐CoV‐2 enters the host cells through the angiotensin‐converting enzyme 2 (ACE‐2) receptor.^3^ In this regard, after binding of virus Spike protein to the receptor, the virus enters the host cells. ACE‐2 is involved in SARS‐CoV‐2 by entering the cells and finally cytokine storms.^3^ In general, few studies have been performed on the expression of ACE‐2 in patients with COVID‐19, and the results are slightly contradictory.^4^, ^5^ There are also very few studies on the mechanisms of regulation of ACE‐2 expression in these patients. Some polymorphisms, methylation, and microRNAs are the three mechanisms in the expression regulation of the different genes; therefore, in this study, we decided to evaluate the role of the mentioned mechanisms in regulating the ACE‐2 gene expression among the COVID‐19 patients.

Based on our results, hypo‐methylation was the vital mechanism in the regulation of expression of this gene. Studies on various organisms, including humans, have suggested roles for nutrient metabolism in regulating the epigenetic state in normal and disease states.^6^, ^7^ Accordingly, in this study, the effect of the amount of B9 and B12 vitamins, which are present in natural foods, are involved in the one‐carbon metabolism cycle, and play an essential role in the methylation of various genes, was investigated in the ACE‐2 gene methylation pattern.

## 2 METHODS

### 2.1 Sample collection

Whole blood samples were obtained from COVID‐19 patients at Velayat Hospital and Khatam Pathobiology and Genetics lab, Qazvin, Iran. These patients were divided into two groups: The first group were people with mild symptoms who did not need to be hospitalized, and only their PCR test was positive (*n* = 70). The second group had acute respiratory distress syndrome (ARDS) and were hospitalized in the intensive care unit (ICU; *n* = 70). *n* = 120 samples were taken as controls whose PCR test was negative and had no clinical signs. The patients were informed about the sample collection process and had signed informed consent forms. The recruited patients were men between 50 and 55 years old. Patients with obesity, diabetes, hypertension, heart, kidney, and lung diseases were not included in the study due to many factors involved in the expression and methylation of ACE‐2 gene. The study was approved by the Ethics Committee of Qazvin University of Medical Sciences (Qazvin, Iran).

### 2.2 RNA extraction and QRT‐PCR for ACE‐2 expression detection

RNAs were extracted using RNeasy Mini Kit (Qiagen) and were frozen at −80°C. We used Nano‐Drop 2000c (Thermo) to evaluate the quality and quantity of isolated total RNAs. In this regard, RNA samples with A260/A280 ratios of >1.8 were selected for quantitative analysis. First‐strand complementary DNA (cDNA) synthesis was initially performed using the Revert Aid First Strand cDNA Synthesis Kit (Thermo Scientific, Fermentas). Then, real‐time quantification was performed using Rotor gene‐Q real‐time PCR system (Qiagen). Each real‐time PCR (10 μL) included 1 μL of reverse and forward primers (Exiqon), 5 μL of Ampliqon real Q plus 2× master mix green (Ampliqon), and 4 μL of diluted cDNA. Primer sequences for the ACE‐2 gene were F: 5′‐TCCATTGGTCTTCTGTCACCCG‐3′, R: 5′‐AGACCATCCACCTCCACTTCTC‐3′. The reactions were incubated in a 72‐well optical strip at 95°C for 15 min (enzyme activation), followed by 95°C for 20 s and 60°C for 60 s (40 cycles). All reactions were run in triplicate. After the reactions, the mean Ct was determined from the triplicate PCRs. We used Ct values to evaluate the expression levels of the ACE‐2 gene. It should be noted that Beta‐actin was the endogenous control gene to normalize RNA contents among different samples. The expression value of the ACE‐2 gene relative to internal controls was determined using the 2^−ΔCt^ method.

### 2.3 ACE‐2 gene polymorphisms evaluation

To analyze the polymorphisms of the ACE‐2 gene, we designed the following primers: rs18236622 5′‐CAT CCC TAT TGG CAG GTT AC ‐3′ as forward primer and 5′‐GAC GGT GCG GTG AGA GTG‐3′ as reverse primer. For rs2097723 polymorphism, these primers were 5′‐CTTTGG GGAGCTGAAGGACTACTA C‐3′ and 5′‐CAC TTT GTG ACC ATT CCG GTT TG‐3′ as forward and reverse primers, respectively. For doing PCR, first, we isolated genomic DNA from the blood cells by using Dyna Bio™ Blood/Tissue DNA Extraction Mini Kit (Takapouzist). Our PCR condition was primary denaturation at 95°C for 5 min, followed by 40 cycles of denaturation at 95°C for 2 min, annealing at 60°C for 1 min, extension at 70°C for 1 min, and a final extension at 70°C for 5 min. After the PCR procedure, we sequenced PCR products based on the Sanger method using ABI 3730XL Capillary Sequencer. The ACE‐2 normal sequences were obtained from NCBI website: http://www.ncbi.nlm.nih.gov, then were assembled by using Chromas software (version 2.4).

### 2.4 DNA extraction and bisulfite modification

The standard phenol‐chloroform method was used to extract DNA from the blood samples.^8^ Then, for the methylation study, 10 ng of genomic DNA was bisulfited using the EpiJET™ Bisulfite Conversion kit (Thermo Fisher Scientific, Inc) according to the kit instructions. Bisulfite treatment converted nonmethylated cytosine ‘C’ bases to thymine ‘T’ bases; however, the methylated cytosine ‘C’ was left unchanged. The methylation levels were determined by using the Pyrosequencing method. In this regard, first, CpG islands of the ACE‐2 gene were identified using MethPrimer (www.urogene.org/methprimer). Then, CpG sites of interest and PCR primers were selected according to the general rules and advice of the primer design. After the bisulfate treatment procedure, the target sequence of the ACE‐2 gene was amplified by polymerase chain reaction with these primers: F 5′‐GGGTAG ATTAAGAGGTTAGAAG‐3′, R 5′‐Biotin‐ATTCACCCC ATTCTCCTA‐3′, and this condition: 95°C for 15 min (Hot start), followed by 35 cycles at 95°C for 20 s (denaturation), 56.5°C for 45 s (annealing), and 72°C for 45 s (extension) Pyrosequencing was also carried out using Pyromark Gold Q96 (Qiagen GmbH) with this primer sequence: 5′‐TTATTA AAAATATAAAAATATTAG‐3′. Subsequently, complete cytosine conversion at a non‐CpG site ensured successful bisulfite conversion. ACE‐2 gene sequence and CpG sites were CTGA**CG**CATCG**CG**TCCCAGT**CG**GGC**CG**A. To evaluate the overall ACE‐2 methylation level in BALF and blood samples, the amount of C relative to the amount of C and T at each CpG site was calculated as the percentage.

### 2.5 QRT‐PCR for microRNA expression evaluation

We used TRIZOL reagent to isolate the total blood RNAs based on kit protocols (Invitrogen Life Technology Co). In order to separate the proteins, three steps of phenol/chloroform purification were performed. To evaluate the concentration and purity of RNA, we used Nano Drop w ND‐1000.

miRCURY LNA Universal RT microRNA kit was used to reverse the transcription of RNA (Exiqon, Denmark). The final volume was 10 μL containing 1 ng/μL of purified total RNA, 5× reaction buffer, Enzyme mix, and nuclease‐free water. The mix was incubated at 42°C for 60 min, then at 95°C for 5 min (for enzyme inactivation). It was then quickly cooled to 4°C. The three miRNAs examined in our present study were *hsa‐miR‐1246* (MIMAT 0005898), *hsa‐miR‐200c* (MIMAT0000617), and *hsa‐miR‐let7‐b* (MIMAT 0000063). Also, we used *hsa‐miR30a‐5p* (MIMAT0000087) and *hsa‐miR100‐5p* (MIMAT 0000102) as internal controls.

Subsequently, real‐time quantification was performed using Rotor gene‐Q real‐time PCR system (Qiagen). Each real‐time PCR (10 μL) included 1 μL of reverse and forward primers (Exiqon), 5 μL of Ampliqon real Q plus 2× master mix green (Ampliqon), and 4 μL of diluted cDNA. The reactions were incubated in a 72‐well optical strip at 95°C for 15 min (enzyme activation), followed by 95°C for 20 s and 60°C for 60 s (40 cycles). All reactions were run in triplicate. After the reactions, the mean Ct was determined from the triplicate PCRs. We used Ct values to evaluate the expression levels of the three miRNAs. Note that hsa‐miR30a‐5p and hsa‐miR100‐5p are the endogenous control genes to normalize RNA contents among different samples. The expression value of miRNAs relative to internal controls was determined using the 2^−ΔCt^ method.

### 2.6 Determination of vitamin B12 and folate in serum

Blood samples were collected from fasting ARDS individuals (since recent food intake may increase the folic acid level appreciably) in 5‐ or 10‐mL evacuated glass tubes. We allowed the blood to clot at room temperature, and the serum was collected by centrifugation. Vitamin B12 and folate were measured in serum samples of patients simultaneously using a vitamin B12/folate RIA kit (IBL). In this kit, B12 vitamin <120 pg/mL is low, 120–160 pg/mL is intermediate, 160–970 pg/mL is normal, and >970 pg/mL is high. Regarding the folate, the normal range is >1.5 ng/mL.

### 2.7 Statically analysis

The results of this research were analyzed by Graph Pad software (GraphPad PRISM V 5.04 analytical software). The difference in the expression and methylation levels of ACE‐2 among the studied samples was calculated with ANOVA one‐way test. The association between different genotypes of the mentioned polymorphisms and disease was assessed by computing the odds ratio and 95% confidence intervals (95% CI) from logistic regression analyses. We also used the Hardy–Weinberg equilibrium test for polymorphism frequency evaluation. For further investigation, the correlation between the prevalence of ACE‐2 expression and methylation was analyzed by Spearman's rank correlation. All *p*‐values were two‐tailed, with *p* < 0.05 considered statistically significant.

## 3 RESULTS

### 3.1 Samples characterization

A total of 140 patients with COVID‐19 (*n* = 70 mild COVID‐19, *n* = 70 ARDS) and 120 controls were recruited. The mean age of the mild COVID‐19 patients was 49.8 ± 7.2 years, and ARDS patients with COVID‐19 were 52.0 ± 8.3 years which were not significantly different from the controls 51.8 ± 4.2 (*p* = 0.116). Symptoms of patients with mild COVID‐19 were fever, cough, tiredness, and loss of smell and taste. ARDS patients had severe shortness of breath or breathlessness, rapid and labored breathing, extreme tiredness, and muscle fatigue admitted to the ICU section. Because age and sex affect the methylation pattern of the ACE‐2 gene, all patients and controls were male. There was no significant difference in their age range and other factors such as diabetes and hypertension.

#### 3.1.1 ACE‐2 expression and methylation level in patients and controls

**FIGURE 1:**
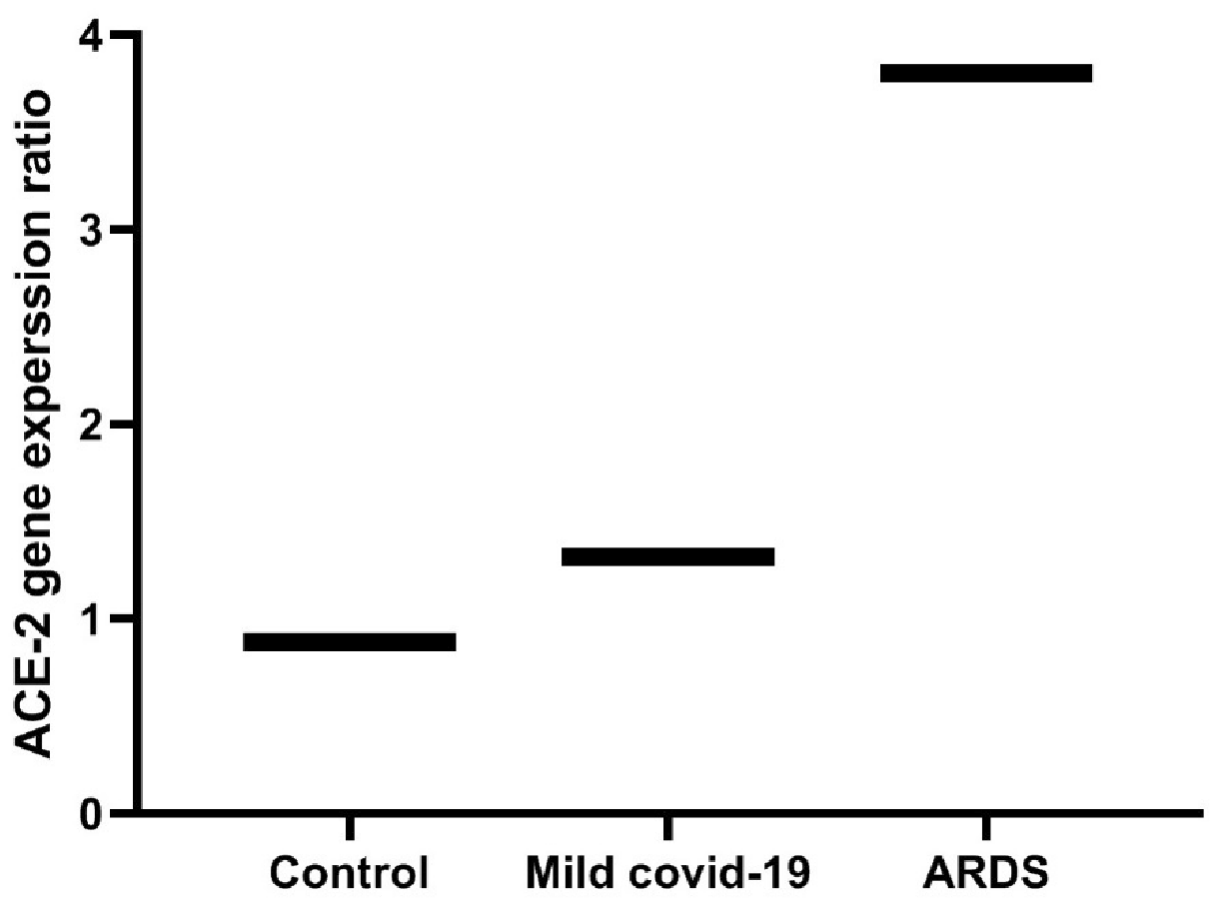
The ACE‐2 expression ratio comparison among the three studied groups, as shown there was a significant over‐expression in ARDS patients in comparison with controls (*p* < 0.00001) and mild COVID‐19 patients (*p* < 0.001).

**FIGURE 2:**
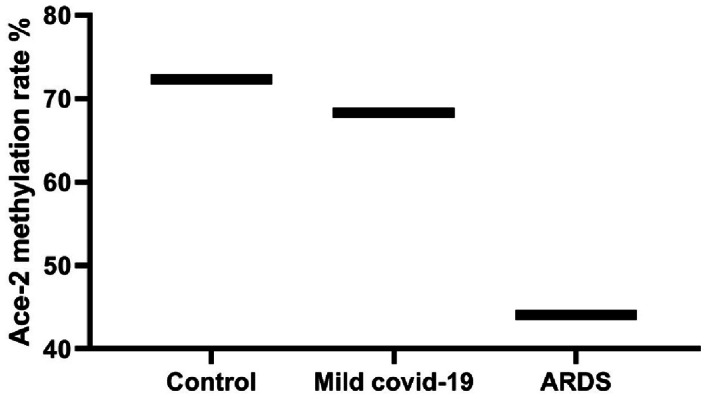
Methylation rate of the ACE‐2 gene between the controls and patients. As it can be seen in the graph, there was a significant hypomethylation rate in ARDS patients (*p* < 0.0001).

ACE‐2 expression level in mild COVID‐19 patients was 1.32 ± 0.88 as compared to controls (0.88 ± 0.12), and there was no significant difference (*p* = 0. 12; Figure 1); although the expression had been increased, this increase was not significant. In ARDS patients, the ACE‐2 levels were significantly increased (3.8 ± 0.77) compared with controls (0.88 ± 0.12; *p* < 0.00001; Figure 1). Also, there was a significant difference in ACE‐2 expression ratio between mild and ARDS patients (*p* < 0.001). We examined the ACE‐2 methylation rate in mild COVID‐19 and ARDS patients compared with non‐COVID‐19 samples. In mild COVID‐19 blood samples, we observed a low rate of ACE‐2 methylation (68.3 ± 3.1; %) in comparison with the matched control samples (72.3 ± 5.1; %), but this difference was not significant (*p* = 0.21; Figure 2). A significant hypo‐methylation of the ACE‐2 gene in ARDS patients (44.07 ± 6.1; %) was observed in comparison with control samples (72.3 ± 5.1; %; *p* < 0.0001; Figure 2). The results of the Spearman correlation showed a significant correlation between the over‐expression of the ACE‐2 gene and hypo‐methylation (*R* = 0.4, *p* = 0.03; Table 3).

#### 3.1.2 Polymorphisms of ACE‐2 gene

In this research, we evaluated the prevalence of ACE‐2 gene polymorphisms among the studied samples. These polymorphisms were rs182366225 C>T and rs2097723 T>C. These two polymorphisms are in the regulatory region of the ACE‐2 gene and cause over‐expression of ACE‐2(9). Data analysis results showed that the frequency of CC, CT, and TT genotypes in rs182366225 polymorphism had no significant difference between mild and ARDS patients in comparison with control samples (*p* > 0.05; Table 1). But significant high frequency of TT genotype was observed in mild COVID‐19 patients in comparison with ARDS patients; this showed a slightly protective effect of this genotype (*p* > 0.01).

**TABLE 1:** Genotypes frequency of the ACE‐2 gene polymorphisms among the studied groups.

ACE‐2 SNPs	Genotypes	Control (*n* = 120)	Mild COVID‐19 (*n* = 70)	ARDS (*n* = 70)
rs182366225	CC	66 (55%)	34 (49.1%) 1 (Ref)	29 (41.7%) 1 (Ref)
	CT	43 (36.2%)	28 (39.2%) 0.9 (0.53–1.7)	27 (39%) 1.3 (0.75–2.3)
	TT	11 (8.8%)	**14** * **(19.3%) 2.5 (1.02–6.1)**	8 (11.7%) 1.5 (0.41–5.6)
	C allele	175 (73%)	96 (68.5%) 1 (Ref)	29 (41.7%) 1 (Ref)
	T allele	65 (27%)	**55** * **(39%) 1.7 (0.95–1.7)**	55 (39%) 1.7 (0.95–1.7)
rs2097723	TT	57 (48%)	34 (49.2%) 1 (Ref)	35 (50.2%) 1 (Ref)
	CT	50 (41.2%)	29 (40.8%) 1.5 (0.83–2.7)	28 (40%) 0.6 (0.3–1.3)
	CC	13 (10.8%)	7 (10%) 0.8 (0.3–2.2)	7 (10.8) 1.09 (0.6–1.9)
	T allele	164 (68.3%)	97 (69.2%) 1 (Ref)	98 (70%) 1 (Ref)
	C allele	76 (31.7%)	43 (30.8) 0.95 (0.52–1.7)	42 (30%) 0.95 (0.52–1.7)

* Shows significant difference within groups compared with control group (*p* < 0.05).

Regarding the rs2097723 T>C polymorphism, there was no a significant difference in CT and CC polymorphisms frequency between mild and ARDS patients in comparison with controls (*p* > 0.05); also, the frequency of TT genotype in mild patients did not have a significant difference in comparison with ARDS patients (*p* = 0.2; Table 1).

#### 3.1.3 Expression of miRNAs

**FIGURE 3:**
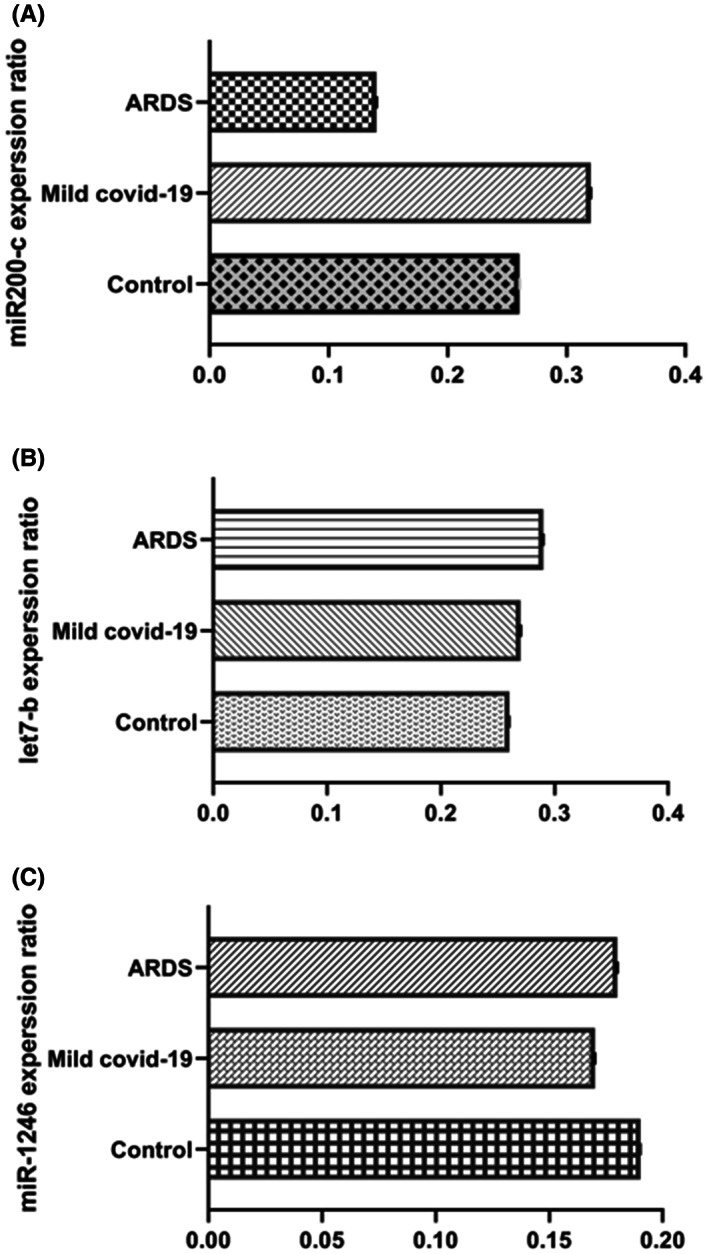
(A) miR‐200c expression rate in the studied samples, as seen miR‐200c significantly downregulated in ADRS patients (*p* < 0.0001). (B) Let‐7b showed no significant difference among the different studied samples (*p* > 0.05). (C) Also there was no a significant difference in the miR‐1246 expression ratio between patients and controls (*p* > 0.05).

The expression of the three studied miRNAs was estimated by qRTPCR. The expression of hsa‐miR‐200c‐3p in the mild COVID‐19 group was (0.26 ± 0.12), which downregulated compared with the control samples (0.32 ± 0.17), but this downregulation was not significant (*p* = 0.12). The results of Tukey's post‐test showed that the expression of this miRNA decreased significantly in ARDS patients (0.14 ± 0.1) compared with controls (0.32 ± 0.17; *p* < 0.0001; Figure 3A).

The ANOVA results indicated that among the mild COVID‐19 patients, the expression of Let7‐b (0.27 ± 0.12), mir‐1246 (0.16 ± 0.12), and mir‐4262 (0.16 ± 0.1) did not have significant difference in comparison with controls (0.26 ± 0.12), (0.19 ± 0.09), (0.29 ± 0.22), and (0.27 ± 0.22; *p* > 0.05; Figure 3A–C). In ARDS patients, we did not observe under‐expression of the studied miRNAs in comparison with controls. Let7‐b (0.24 ± 0.12 vs. 0.26 ± 0.19; *p* = 0.12), mir‐1246 (0.18 ± 0.11 vs. 0.19 ± 0.12; *p* = 0.34), mir‐4262 (0.16 ± 0.1 vs. 0.17 ± 0.13; *p* = 0.45; Figure 3A–C).

### 3.2 Evaluation of polymorphisms frequency, miRNA expression, and ACE‐2 hypo‐methylation rate between two ARDS groups

**FIGURE 4:**
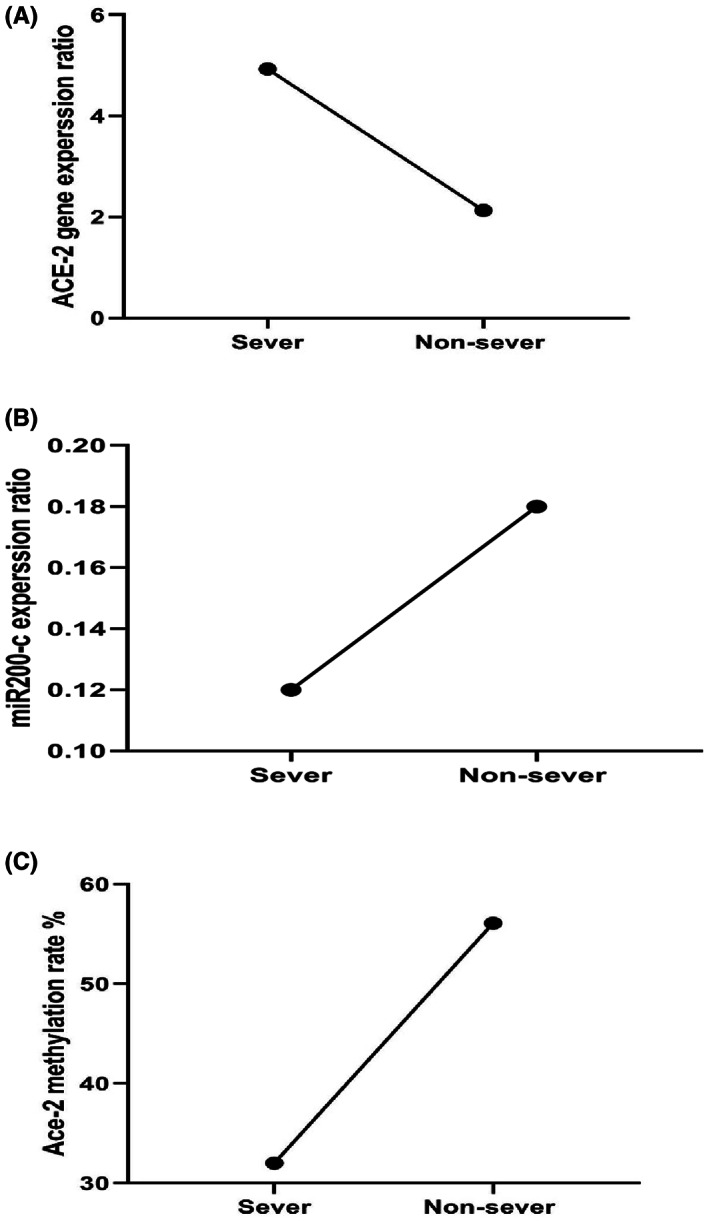
(A) A comparison of ACE‐2 expression rate between severe and nonsevere ARDS patients, as seen ACE‐2 significantly upregulated in severe ARDS patients (*p* < 0.001). (B) Significant downregulation of miR‐200c in severe ARDS patients (*p* < 0.0001). (C) Significant hypomethylation of ACE‐2 gene among the severe ARDS patients (*p* < 0.001).

In another section of the study, we divided ARDS patients into two groups: patients who used only oxygen masks (*n* = 45) and patients who had more severe symptoms and were connected to ventilators and eventually died (*n* = 25). The results of ARDS patients' ACE‐2 expression showed that in severe samples, expression ratio was 4.93 ± 2 ± 0.2 and in nonsevere samples was 2.13 ± 0.98; this difference was significant (*p* < 0.001; Figure 4A). Then, the three factors that effected ACE‐2 gene expression (polymorphism, miRNA expression, and methylation) in these two ARDS groups were evaluated. The frequency of different genotypes of rs182366225 and rs182366225 polymorphisms between two ARDS patient groups showed no significant difference (*p* > 0.05).

Among the studied miRNAs, only miR‐200c‐3p expression had a significant difference between the two ARDS groups. In severe ARDS patients, miR‐200c‐3p was downregulated significantly (0.12 ± 0.12) in comparison with nonsevere ARDS patients (0.18 ± 0.24; *p* < 0.0001; Figure 4B). Regarding the methylation evaluation, the methylation rate was significantly high (56.1 ± 11; %) in nonsevere ARDS patients in comparison with severe ones (32 ± 14; %; *p* < 0.001; Figure 4C).

### 3.3 B12 and folate concentration

**FIGURE 5:**
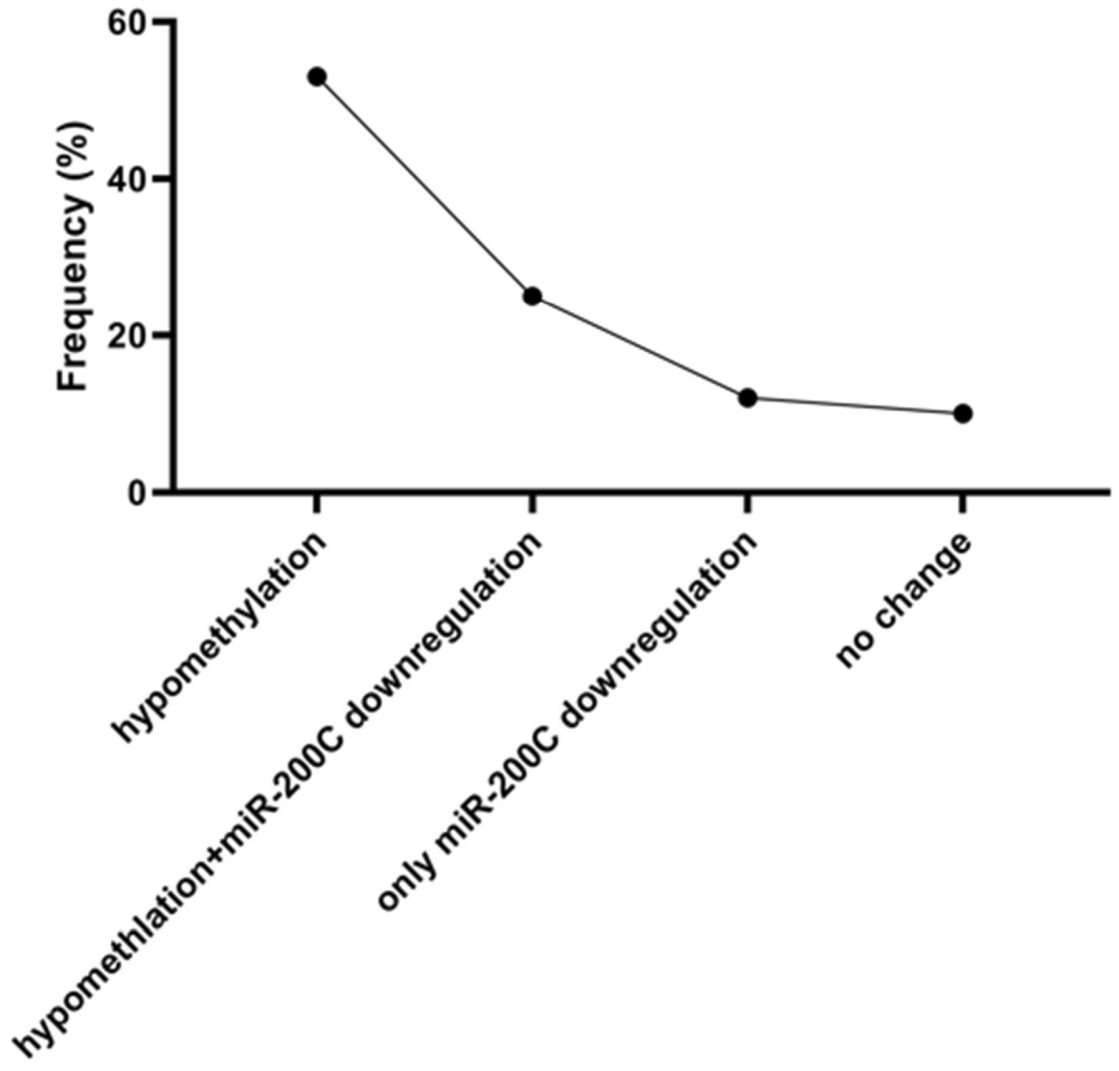
The frequency of different mechanisms involved in ACE‐2 expression. As seen in the figure, the highest percent of ARDS patients showed hypomethylation of the ACE‐2 gene.

The interesting point about this research was that a high percentage of the studied ARDS patients had ACE‐2 gene hypo‐methylation in comparison with miR‐200c‐3p downregulation; so, 53% of ARDS patients had only hypo‐methylation, 25% miR‐200c‐3p downregulation and ACE‐2 hypo‐methylation, 12% only miR‐200c‐3p downregulation, and 10% showed no change (Figure 5). Because environmental factors, such as vitamins, involve in mono‐carbon metabolism and play an important role in the methylation of genes, the amount of B12 and folate vitamins in these individuals was examined. In severe ARDS patients, the concentration of B12 was 127.2 ± 7.8, but in nonsevere patients, it was 152.4 ± 6.3, and there is a significant difference in B12 vitamin concentration between the two ARDS groups (*p* < 0.001). Folate concentration in severe ARDS patients was 2 ± 0.99 and in nonsevere was 8.8 ± 0.99, and this difference was significant (*p* < 0.01; Table 2). There was a significant positive correlation between hypo methylation rate and B12, folate deficiency (*R* = 0.32, *p* < 0.001 and *R* = 0.37, *p* < 0.000, respectively; Table 3).

**TABLE 2:** Comparison of B12 and B9 vitamins level in serums of studied samples.

	Severe ARDS	Nonsevere ARDS	Normal range	*p*‐value
B12 concentration pg/mL	127 ± 7.8	152.4 ± 6.3	120–160	<0.001
Folate concentration ng/mL	2 ± 0.99	8.8 ± 1.2	>1.5	<0.01

**TABLE 3:** Correlation of ACE‐2 hypomethylation rate with ACE‐2 expression, B12 and folate deficiency among the studied patients.

	Ace‐2 expression	B12 deficiency	Folate deficiency
Hypo methylation rate			
Pearson correlation	0.4	0.32	0.37
Sig. 2 tailed	0.03	<0.001	0 < 0001
*N*	140	140	140

	Severe ARDS	Nonsevere ARDS	Normal range	*p*‐value
B12 concentration pg/mL	127 ± 7.8	152.4 ± 6.3	120–160	<0.001
Folate concentration ng/mL	2 ± 0.99	8.8 ± 1.2	>1.5	<0.01

## 4 DISCUSSION

This research showed the critical role of B vitamins deficiency in hypo‐methylation, over‐expression of the ACE‐2 gene, and its role in the severity of ARDS patients. Various studies have recently been performed on the role of the ACE‐2 receptor in the entry of the coronavirus into host cells which reveal an interplay of genetic and epigenetic alterations in host response.^9^, ^10^ Angiotensin‐converting enzymes (ACEs) play an essential role in the regulation of blood pressure as well as electrolyte and fluid homeostasis. These enzymes are parts of the renin‐angiotensin (Ang) system. The function of ACE is the cleavage of AngI into AngII. Then, AngII degrades into small peptides by the ACE‐2 enzyme.^3^, ^4^ SARS‐CoV‐2 enters the host cells through the ACE‐2 receptor.^4^

Our results showed over‐expression of the ACE‐2 gene in ARDS patients; in line with our study, Sawalha et al.^13^ showed over‐expression of the ACE‐2 gene in the T cells of lupus patients with COVID‐19.^11^ In another study, Gerad et al. (2020) showed ACE‐2 upregulation in the lung tissue and serum of COVID‐19 ARDS patients.^12^ Likewise, Bruna et al. (2020) indicated a high‐expression of ACE‐2 in lung tissue of severe COVID‐19 patients with comorbidities.^13^ Since studies have shown the role of increased expression of the ACE‐2 gene in disease severity, it is essential to investigate the mechanism of increased expression for treatment. In this research, frequency of some ACE‐2 gene polymorphisms, its methylation pattern, and expression of some miRNAs that involved in ACE‐2 expression regulation were investigated. The studied polymorphisms were rs182366225 C>T and rs2097723 T>C. The role of these polymorphisms in the over‐expression of the ACE‐2 gene and their high frequency in the Asian population was shown.^14^ In our studied population, there was no significant difference in the frequency of the mentioned polymorphisms between patients and controls.

In another section of our research, we evaluated the expression rate of some miRNAs which regulate the ACE2 expression. Based on our results, only miR‐200c expression was significantly downregulated in ARDS patients. Different studies showed that various miRNAs control ACE‐2 expression, and they emphasize the critical role of miR‐200c‐3p in the regulation of ACE‐2 expression.^15^, ^16^, ^17^, ^18^ There are contradictory results regarding increasing and decreasing the expression of this miRNA in COVID‐19 patients. Some studies showed upregulation of miR‐200c‐3p and downregulation of ACE‐2 gene^13^ and some other studies showed opposite results.^13^ MiR‐200c act as tumor suppressor or oncogene in certain types of cancers and serve as a suitable prognostic marker for patients with cancer.^19^ Research studies have recently implicated DNA methylation in the regulation of the ACE‐2 gene expression, indicating that the host epigenome may represent a risk factor for COVID‐19 infection.^20^, ^21^ About the methylation pattern evaluation of the ACE‐2 gene, our results showed hypomethylation of this gene in ARDS patients. In line with our study, Sawalha et al.,^13^ based on whole‐genome DNA methylation data, found hypo‐methylation and over‐expression of the ACE‐2 gene in the T cells of lupus patients with COVID‐19. They concluded that oxidative stress induced by viral infections could strengthen the DNA methylation defect in lupus, leading to further ACE‐2 hypo‐methylation and enhanced viremia.^11^ In another study, the reasons for low involvement of children with COVID‐19, high methylation rate, and low expression of ACE‐2 gene are reported.^22^

**FIGURE 6:**
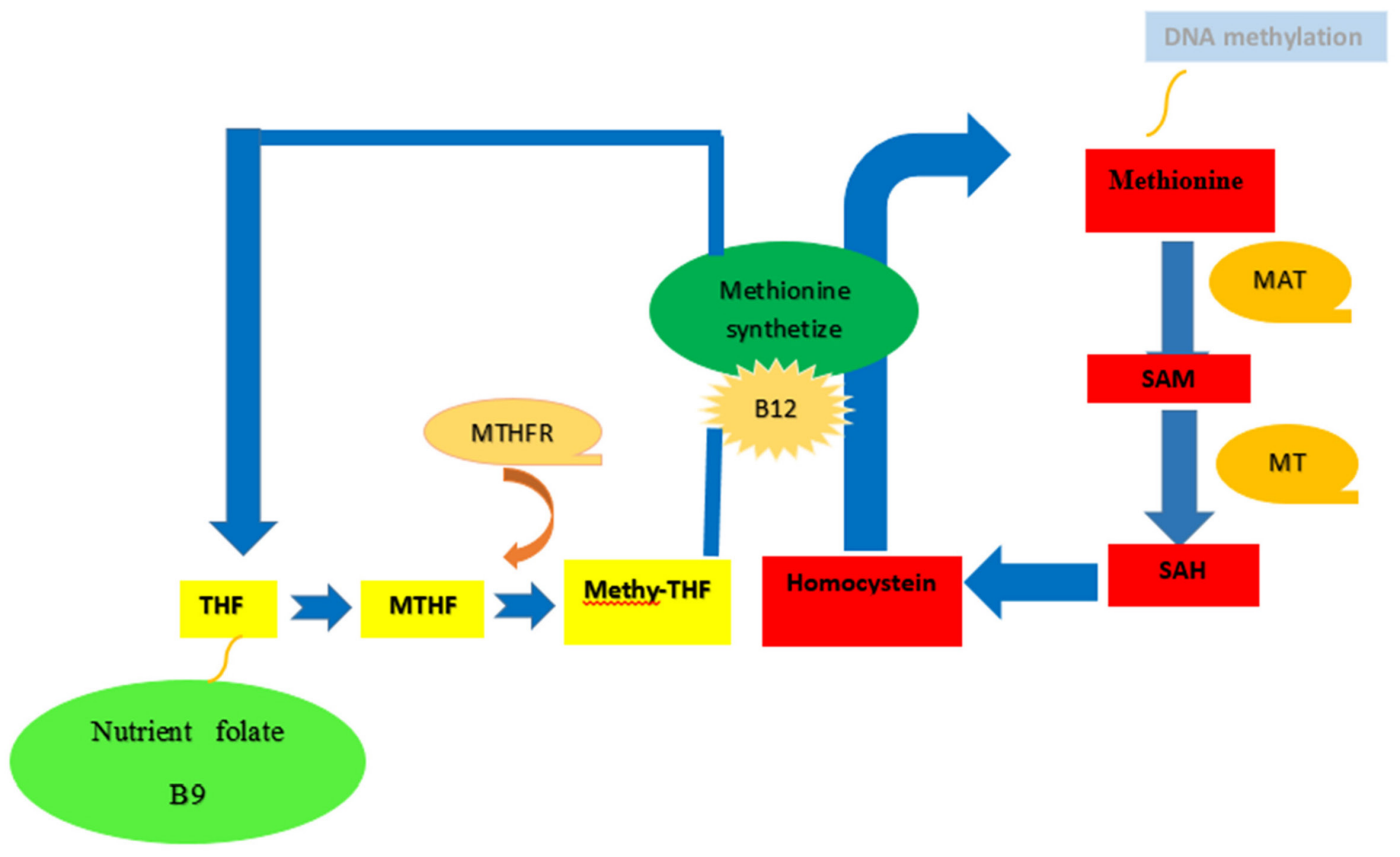
The folate and methionine cycles are interconnected and are required for many cellular processes, and the methionine from this cycle provides the methyl group needed for the methylation of various genes. As seen in this diagram, B9 and B12 are two important components of these cycles. A simplified schematic drawing of the folate and methionine cycles. Metabolites and enzymes: MAT, methionine adenosyl transferees; MT, methyl tansferase; MTHF, Methylene tetra hydro folate; SAH, S‐adenosylhomocysteine; SAM, S‐adenosyl methionine; THF, tetra hydro folate.

The interesting point of this study, which seems to have not been reported so far, is to investigate the effect of B vitamins on the methylation pattern of the ACE‐2 gene. In general, nutrient B vitamins, by involving in the one‐carbon metabolism cycle, would provide the methyl group needed for gene methylation. In this regard, dietary folate is converted to dihydrofolate (DHTF) and then tetrahydrofolate (TH3). The primary methyl group donor for DNA methylation reactions is 5‐methyl‐tetrahydrofolate (CH3‐THF), required for the transformation of homocysteine into methionine mediated by methionine synthase with vitamin B12 as a co‐substrate leading to the synthesis of S‐adenosyl methionine (SAM); SAM is considered the universal methyl donor and is used by methyltransferases to methylate metabolites, RNA, DNA, and proteins, including histones^23^, ^24^ (Figure 5). Various reports have shown the role of one‐carbon metabolism in epigenetics.^25^, ^26^, ^27^ Based on our results for the first time we showed, deficiency of B_9_ and B_12_ vitamins has an important role in hypo‐methylation and over‐expression of the ACE‐2 gene and severity of symptoms of the COVID‐19 disease. There has been no report regarding this finding, and it seems that taking B vitamins, which are involved in monocarbon metabolism, can play a vital role in reducing the symptoms of COVID‐19. A few reports have shown that taking B vitamins can reduce the symptoms of COVID‐19,^28^ but the exact mechanism has not been discussed. In conclusion, the results of this study showed that among the various mechanisms involved in regulating the ACE‐2 expression (such as polymorphism, miRNAs, and methylation), the reduction of methylation plays a critical role in the overexpression of this gene and the severity of symptoms; moreover, there was a significant and direct correlation between B_9_ and B_12_ deficiency with hypomethylation and overexpression (Figure 6).

## AUTHOR CONTRIBUTION

Reza Najafipour: Design of project, Monitoring of project, and Executor of project. DavoodMohammadi: Sampling and collaboration in writing the manuscript. AbdolmaboodMomeni: Doing the techniques. SaharMoghbelinejad: Design of project, Monitoring of project, and Executor of project and writing the manuscript.

## FUNDING INFORMATION

This research was supported by the Deputy of research and technology of Qazvin University of Medical Science. This work was supported by Qazvin University of Medical sciences [grant number 761]. The funding source has no involvement in the study.

## CONFLICT OF INTEREST STATEMENT

The authors declare no conflicts of interest for this work.

## Data Availability

The data that support the findings of this study are available on request from the corresponding author [SM]. The data are not publicly available because they contain information that could compromise the privacy of research participants.
